# Natural Product Chemistry of Gorgonian Corals of Genus *Junceella*—Part II

**DOI:** 10.3390/md9122773

**Published:** 2011-12-19

**Authors:** Yang-Chang Wu, Jui-Hsin Su, Tai-Ting Chou, Yin-Pin Cheng, Ching-Feng Weng, Chia-Hung Lee, Lee-Shing Fang, Wei-Hsien Wang, Jan-Jung Li, Mei-Chin Lu, Jimmy Kuo, Jyh-Horng Sheu, Ping-Jyun Sung

**Affiliations:** 1 Graduate Institute of Integrated Medicine, College of Chinese Medicine, China Medical University, Taichung 404, Taiwan; Email: yachwu@mail.cmu.edu.tw; 2 Natural Medicinal Products Research Center and Center for Molecular Medicine, China Medical University Hospital, Taichung 404, Taiwan; 3 Institute of Marine Biotechnology, National Dong Hwa University, Pingtung 944, Taiwan; Email: x2219@nmmba.gov.tw (J.-H.S.); cfweng@mail.ndhu.edu.tw (C.-F.W.); chlee016@mail.ndhu.edu.tw (C.-H.L.); jinx6609@nmmba.gov.tw (M.-C.L.); jimmy@nmmba.gov.tw (J.K.); 4 National Museum of Marine Biology and Aquarium, Pingtung 944, Taiwan; Email: whw@nmmba.gov.tw (W.-H.W.); jj@nmmba.gov.tw (J.-J.L.); 5 Uni-President Biotech Co., LTD., No. 31, Gongye 2nd Rd., Annan District, Tainan 709, Taiwan; Email: tong.xin@msa.hinet.net (T.-T.C.); pin@unibiotech.com.tw (Y.-P.C.); 6 Department of Life Science and Institute of Biotechnology, National Dong Hwa University, Hualien 974, Taiwan; 7 Department of Sport, Health and Leisure, Cheng Shiu University, Kaohsiung 833, Taiwan; Email: lsfang@csu.edu.tw; 8 Department of Marine Biotechnology and Resources, National Sun Yat-sen University, Kaohsiung 804, Taiwan; 9 Division of Marine Biotechnology, Asia-Pacific Ocean Research Center, National Sun Yat-sen University, Kaohsiung 804, Taiwan

**Keywords:** *Junceella*, gorgonian, briarane, Indo-Pacific Ocean, South China Sea

## Abstract

The structures, names, bioactivities, and references of 81 new secondary metabolites obtained from gorgonian corals belonging to the genus *Junceella* are described in this review. All compounds mentioned in this review were obtained from sea whip gorgonian corals *Junceella fragilis* and *Junceella juncea*, collected from the tropical and subtropical Indo-Pacific Ocean.

## 1. Introduction

This review describes 81 new natural products from gorgonian corals belonging to the genus *Junceella* (phylum Cnidaria, class Anthozoa, order Gorgonacea, family Ellisellidae) [1,2,3,4]. Extending from a previous review in 2004 [5], this review describes compounds reported from November 2003 to September 2011 and provides structures, names, bioactivities and references for all compounds in tabular form. 

## 2. Natural Products from Gorgonian Corals Belonging to the Genus *Junceella*

### 2.1. *Junceella fragilis*

Two new chlorinated briarane-type diterpenoids (3,8-cyclized cembranoids), (−)-2-deacetyl-junceellin (**1**) and (−)-3-deacetyljunceellin (**2**) (Table 1), along with five known briaranes, junceellin, praelolide, and junceellolides A, B and D, were isolated from the gorgonian *J. fragilis*, collected at the Pass Reef of Madang, Papua New Guinea [6]. The absolute stereochemistry of (−)-3-deacetyljunceellin (**2**) was determined by the application of a new method using a combination of proton chemical shifts and molecular dynamic calculation. 

**Table 1:** The new natural products from *Junceella fragilis*-I.

Structure	No.	Name	Biological Activity	Ref.
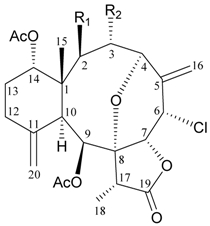	**1**	(−)-2-Deacetyljunceellin (R_1_ = OH, R_2_ = OAc)	n.r. *^a^*	[6]
	**2**	(−)-3-Deacetyljunceellin (R_1_ = OAc, R_2_ = OH)	n.r.	[6]

*^a^* n.r. = not reported.

During the past 30 years, a series of interesting and bioactive natural products has been isolated from various marine invertebrates collected off the South China Sea [7,8]. Five new briaranes, junceellonoids A–E (**3**–**7**) (Table 2) [9,10], eight known briaranes, junceellins A and B, junceellolides A–D, umbraculolide A and praelolide, along with three known steroids, 24α-methylcholest-7,22-dien-3β,5α,6β-triol, cholestan-3-ol and cholesterol, were isolated from *J. fragilis* inhabiting the South China Sea [9,10,11,12]. Junceellonoids C (5) and D (6) exhibited cytotoxicity toward human breast carcinoma MDA-MB-231 and MCF-7 cells [10]. 

**Table 2:** The new natural products from *Junceella fragilis*-II.

Structure	No.	Name	Biological Activity	Ref.
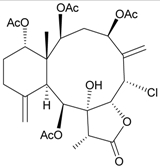	**3**	Junceellonoid A	n.r. *^a^*	[9]
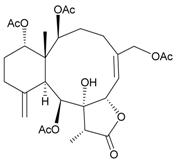	**4**	Junceellonoid B	n.r.	[9]
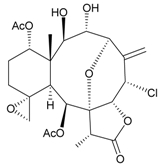	**5**	Junceellonoid C	exhibited cytotoxicity toward MDA-MB-231 and MCF-7 cells at a concentration of 100 µM	[10]
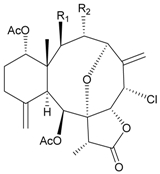	**6**	Junceellonoid D (R_1_ = R_2_ = OH)	exhibited cytotoxicity toward MDA-MB-231 and MCF-7 cells at a concentration of 100 µM	[10]
	**7**	Junceellonoid E (R_1_ = R_2_ = OAc)	n.r.	[10]

*^a^* n.r. = not reported.

In continuing research on the new substances obtained from gorgonian corals distributed in the waters of Taiwan at the intersection of the Kuroshio current and the South China Sea surface current, the gorgonian *J. fragilis* was studied to examine the properties of its organic extract. Thirty-one new briaranes, 9-*O*-deacetylumbraculolide A (**8**) [13], junceellolides H–L (**9**–**13**) [14,15,16], fragilides A–J (**14**–**23**) [17,18,19,20,21,22,23,24] and frajunolides A–O (**24**–**38**) [25,26,27] (Table 3); 16 known briaranes, prarelolide [14,26,28], junceellin A [12,14,26,28], (1*R*,2*R*,5*Z*,7*R*,8*S*,9*R*,10*R*,12*R*,14*R*,17*S*)-2,14-diacetoxy-8,17-epoxide-9,12-dihydroxybriara-5,11(20)-dien-19-one [15], (−)-11β,20β-epoxy-4-deacetoxyjunceellolide D [16,25,26,29], junceellonoid D [22], juncins Y, Z and ZI [22,26], (+)-11β,20β-epoxyjunceellolide D [23,29], junceellolides A–E and K [25,26], and umbraculolide A [25,26]; and three known steroids, ergosterol peroxide [26], deoxycholic acid 3,12-diacetate, and deoxycholic acid 3,12-diacetate methyl ester [30], were isolated from *J. fragilis* collected off the waters of Taiwan. The structure, including the absolute configuration, of junceellolide J (**11**) was confirmed by single-crystal X-ray diffraction analysis and chemical conversion [16]. Fragilide A (**14**) was the first briarane derivative found to possess a 6-hydroxy group [17]. The geometry of the Δ^3,5(16)^-butadiene system in fragilide B (**15**) was found to be of an *s*-*cis* form [18]. The ^13^C NMR data for the known briaranes praelolide and junceellin were reassigned by 2D NMR experiments [14].

**Table 3:** The new natural products from *Junceella fragilis*-III.

Structure	No.	Name	Biological Activity	Ref.
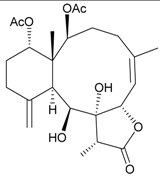	**8**	9-*O*-Deacetylumbraculolide A	n.r. *^a^*	[13]
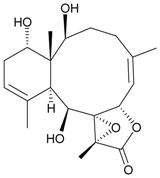	**9**	Junceellolide H	not active in cytotoxicity testing with P-388D1, DLD-1, IMR-32, RPMI 7951 and CCRF-CEM tumor cells *^b^*	[14]
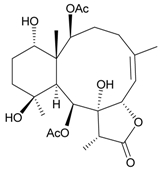	**10**	Junceellolide I	n.r.	[15]
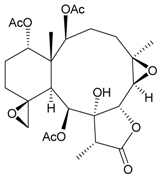	**11**	Junceellolide J	not active in anti-inflammatory bioassay	[16]
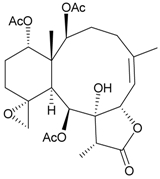	**12**	Junceellolide K	weakly anti-inflammatory	[16]
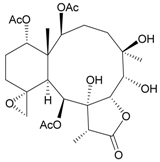	**13**	Junceellolide L	not active in anti-inflammatory bioassay	[16]
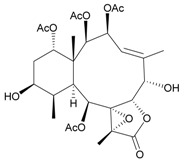	**14**	Fragilide A	n.r.	[17]
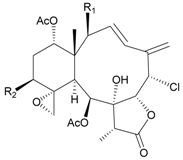	**15**	Fragilide B (R_1_ = OC(O)CH_2_CH_3_, R_2_ = H)	weakly anti-inflammatory	[18]
	**20**	Fragilide G (R_1_ = R_2_ = OAc)	not active in cytotoxicity testing with DLD-1 and CCRF-CEM cells	[22]
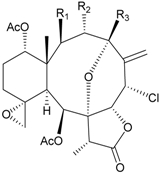	**16**	Fragilide C (R_1_ = OCOCH_2_CH_3_, R_2_ = H, R_3_ = OH)	weakly anti-inflammatory	[19]
	**23**	Fragilide J (R_1_ = OH, R_2_ = OAc, R_3_ = H)	weakly anti-inflammatory	[24]
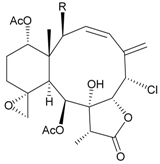	**17**	Fragilide D (= Frajunolide G) (R = OC(O)CH_2_OC(O)CH_2_CH(CH_3_)_2_)	n.r.	[20,26]
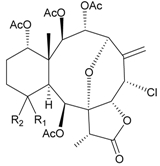	**18**	Fragilide E (R_1_ = β-OH, R_2_ = α-CH_2_OAc)	weakly anti-inflammatory	[21]
	**19**	Fragilide F (R_1_ = α-OH, R_2_ = β-CH_2_Cl)	not active in cytotoxicity testing with DLD-1 and CCRF-CEM cells	[22]
I 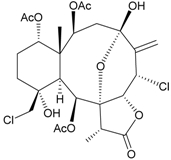	**21**	Fragilide H	not active in cytotoxicity testing with P-388D1, DLD-1, HL-60 and CCRF-CEM cells *^b^*	[23]
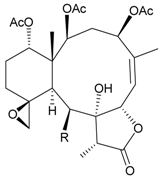	**22**	Fragilide I (R = OC(O)CH_2_CH(CH_3_)_2_)	not active in cytotoxicity testing with P-388D1, DLD-1, HL-60 and CCRF-CEM cells	[23]
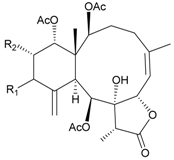	**24**	Frajunolide A (R_1_ = α-OAc, R_2_ = H)	weakly anti-inflammatory	[25]
	**25**	Frajunolide B (R_1_ = α-OAc, R_2_ = OAc)	weakly anti-inflammatory	[25]
	**28**	Frajunolide E (R_1_ = H, R_2_ = OAc)	frajunolides E, J and L were weakly anti-inflammatory	[26]
	**33**	Frajunolide J (R_1_ = α-OC(O)Et, R_2_ = H)	frajunolides E and J were not active in cytotoxicity testing with Hep2, Doay, WiDr and Hela cells *^b^*	[26]
	**35**	Frajunolide L (R_1_ = β-OAc, R_2_ = H)		[27]
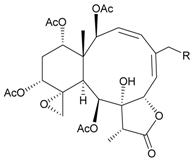	**26**	Frajunolide C (R = Cl)	weakly anti-inflammatory	[25]
	**27**	Frajunolide D (R = OAc)	not active in anti-inflammatory bioassay	[25]
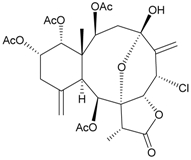	**29**	Frajunolide F	weakly anti-inflammatory	[26]
			not active in cytotoxicity testing with Hep2, Doay, WiDr and Hela cells	
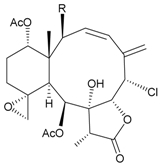	**30**	Frajunolide G (= Fragilide D) (R = OC(O)CH_2_OC(O)CH_2_CH(CH_3_)_2_)	not active in anti-inflammatory bioassay	[20,26]
			not active in cytotoxicity testing with Hep2, Doay, WiDr and Hela cells	
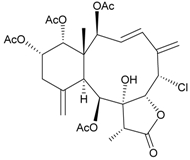	**31**	Frajunolide H	not active in anti-inflammatory bioassay	[26]
			not active in cytotoxicity testing with Hep2, Doay, WiDr and Hela cells	
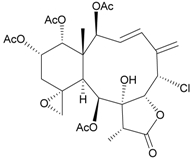	**32**	Frajunolide I	weakly anti-inflammatory	[26]
			not active in cytotoxicity testing with Hep2, Doay, WiDr and Hela cells	
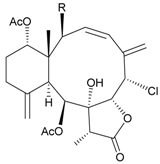	**34**	Frajunolide K (R = OC(O)CH_2_OC(O)CH_2_CH(CH_3_)_2_)	not active in anti-inflammatory bioassay	[26]
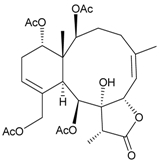	**36**	Frajunolide M	weakly anti-inflammatory	[27]
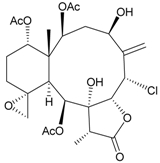	**37**	Frajunolide N	modestly anti-inflammatory	[27]
			not active in cytotoxicity testing with Hep2, Doay, WiDr and Hela cells	
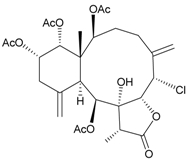	**38**	Frajunolide O	weakly anti-inflammatory	[27]

*^a^* n.r. = not reported; *^b^* P388D1 (mouse lymphoid neoplasm), DLD-1 (human colon adenocarcinoma), IMR-32 (human neuroblastoma), RPMI 7951 (human malignant melanoma), CCRF-CEM (human T-cell acute lymphoblastic leukemia), HL-60 (human promyelocytic leukemia), Hep2 (human liver carcinoma), Doay (medulloblastoma), WiDr (human colon adenocarcinoma), Hela (human cervical epitheloid carcinoma).

In order to determine the stereochemistry of briaranes possessing an exocyclic 11,20-epoxy group, the ^13^C NMR data of the exocyclic 11,20-epoxy groups have been summarized; these appeared at δ_C_ 62–63 and 58–60 ppm, respectively, when the epoxy group existed in the 11S* form and led the cyclohexane rings to exhibit a twist boat conformation. If the epoxy group was in an 11R* configuration, the ^13^C NMR data for C-11 and C-20 appeared at δ_C_ 55–61 and 47–52 ppm, respectively, and the cyclohexane rings were in a chair conformation [16]. The 11,20-epoxybriaranes were only obtained from gorgonian corals belonging to the Ellisellidae family, and, thus compounds of this type could be a chemical marker for gorgonian corals belonging to the Ellisellidae family [31].

From the characteristics of the chemical shifts, it was shown that the briarane derivatives contained an exocyclic double bond between C-11/12. The proton chemical shifts were summed up for the olefin protons H_2_-20; these appear at δ_H_ 4.95–5.30 and 4.85–5.15 ppm, respectively, when the cyclohexane rings are in a twist boat conformation. Likewise, the ^1^H NMR data for H_2_-20 appear at δ_H_ 4.95–5.10 and 4.40–4.75, if the cyclohexane rings were found to exist in a chair conformation [22].

Symbiotic algae (zooxanthella) exist throughout the life cycle of *J. fragilis*, while *J. juncea* is a gorgonian coral free of zooxanthellae [32]. Two known chlorine-containing briaranes, junceellin and praelolide, were isolated in the same proportions from both *J. fragilis* and *J. juncea*, and this observation suggests that junceellin and praelolide could be chemical markers that enable one to infer that the briarane-type compounds are originally synthesized by the host corals [28] and are not produced by their zooxanthella.

In biological activity experiments, the new briaranes, junceellolide K (**12**) [16], fragilides B, C, E and J (**15**, **16**, **18**, **23**) [18,19,21,24], frajunolides A–C (**24**–**26**), E (**28**), F (**29**), I (**32**), J (**33**), L–O (**35**–**38**) (Table 3), and the known compounds (−)-11β,20β-epoxy-4-deacetoxyjunceellolide D [16,25], junceellolide E [25] and umbraculolide A [25], displayed anti-inflammatory activity [33]. Juncin Z was found to exhibit cytotoxicity toward CCRF-CEM cells [22]. 

### 2.2. *Junceella juncea*

Five new steroidal glycosides, 4′-*O*-acetyl-3-*O*-[β-D-arabino-pyranosyl-oxy]-cholest-5-ene-3β,19-diol (**39**) [34] and junceellosides A–D (**40**–**43**) [35], and a new glycerol, 1,2-*O*-[2′-hydroxyoctadecyl]-glycerol (**44**) [34] (Table 4) along with various known metabolites, including four sterols, 24α-methylcholest-7,22-dien-3β,5α,6β-triol, 24α-methylcholest-3β,5α,6β-triol-25-monoacetate, 24α-methylcholest-3β,5α,6β-triol, and 24α-methylcholest-5,23-dien-3β-ol; six amines, 1-*O*-β-D-gluco-pyranosyl-(2*S*,3*S*,4*R*,8*Z*)-2-*N*-(2′-hydroxypalmitoyl)-octadecasphinga-8-ene, (2*S*,3*R*)-2-*N*-palmitoyl-octadecasphinga, (2*S*,3*R*,4*E*)-2-*N*-palmitoyloctadecasphinga-4-ene, thymine, uracil, and adenosine; and batyl alcohol, were isolated from the gorgonian coral *J. juncea*, collected off the South China Sea in 2004–2005 [34,35]. 

**Table 4:** The new natural products from *Junceella juncea*-IV.

Structure	No.	Name	Ref.
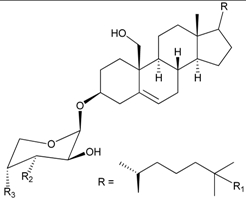	**39**	4′-*O*-Acetyl-3-*O*-[β-D-arabino-pyranosyl-oxy]-cholest-5-ene-3β,19-diol (R_1_ = H, R_2_ = OH, R_3_ = OAc)	[34]
	**40**	Junceelloside A (R_1_ = R_2_ = OH, R_3_ = OAc)	[35]
	**41**	Junceelloside B (R_1_ = R_3_ = OH, R_2_ = OAc)	[35]
	**42**	Junceelloside C (R_1_ = OAc, R_2_ = R_3_ = OH)	[35]
	**43**	Junceelloside D (R_1_ = R_2_ = R_3_ = OH)	[35]
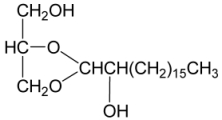	**44**	1,2-*O*-[2′-Hydroxyoctadecyl]-glycerol	[34]

In addition, 14 new briarane derivatives, juncins O–Q (**45**–**47**) [36], R–ZI (**48**–**57**) [37], and ZII (**58**) [38] (Table 5), along with eight known briaranes, praelolide, junceellin, gemmacolides A–C and F, junceellolide D [34,38], and (+)-11β,20β-epoxyjunceellolide D [30,38], were also isolated from *J. juncea*. 

**Table 5:** The new natural products from *Junceella juncea*-V.

Structure	No.	Name	Biological Activity	Ref.
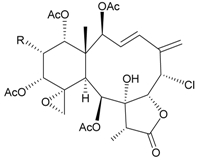	**45**	Juncin O (R = OC(O)CH_2_CH(CH_3_)_2_)	juncins O–Q showed medium antifeedant activity (90.7, 69.0, 46.5%) toward the second-instar larvae of *Spodoptera litura* at a concentration of 500 µg/mL	[36,38]
			juncins O–Q and ZII were not active in cytotoxicity testing with K562, A549, Hela and Hep2 cells *^a^*	
			medium cytotoxicity (cell mortality: 8.7% in 24 h and 11.9% in 48 h) toward the second-instar larvae of *S. litura* at a concentration of 100 µg/mL	
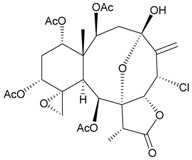	**46**	Juncin P	medium cytotoxicity (cell mortality: 25.3% in 24 h and 29.7% in 48 h) toward the second-instar larvae of *S. litura* at a concentration of 100 µg/mL	[36,38]
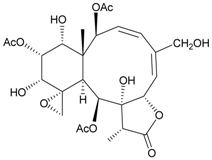	**47**	Juncin Q	medium cytotoxicity (cell mortality: 31.3% in 24 h and 44.0% in 48 h) toward the second-instar larvae of *S. litura* at a concentration of 100 µg/mL	[36,38]
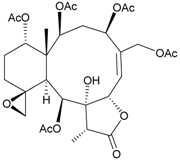	**54**	Juncin X		[37]
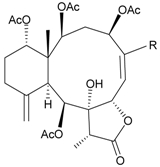	**55**	Juncin Y (R = CH_2_OAc)		[37]
	**56**	Juncin Z (R = CO(O)CH_3_)		[37]
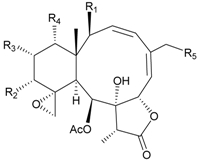	**48**	Juncin R (R_1_ = R_2_ = R_3_ = OAc, R_4_ = OC(O)CH_2_CH(CH_3_)_2_, R_5_ = Cl)	juncins R–ZII (**48**–**58**) exhibited antifouling activity toward the barnacle *Balanus amphitrite* larvae (EC_50_ = 0.004, 0.3, 2.7, 1.6, 3.8, 21.1, 0.004, 0.1, 1.5, 0.5 and 0.004 µg/mL)	[37]
	**49**	Juncin S (R_1_ = R_3_ = R_4_ = OAc, R_2_ = OC(O)CH_2_CH(CH_3_)_2_, R_5_ = Cl)		[37]
	**50**	Juncin T (R_1_ = OC(O)CH_2_OC(O)(CH_2_)_2_CH(CH_3_)_2_, R_2_ = R_3_ = R_4_ = OAc, R_5_ = OH)		[37]
	**51**	Juncin U (R_1_ = R_2_ = R_4_ = OAc, R_3_ = OC(O)CH_2_CH(CH_3_)_2_, R_5_ = OCH_3_)		[37]
	**52**	Juncin V (R_1_ = R_3_ = OAc, R_2_ = R_4_ = OH, R_5_ = OCH_3_)		[37]
	**53**	Juncin W (R_1_ = R_3_ = R_5_ = OAc, R_2_ = R_4_ = OH)		[37]
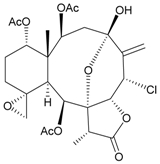	**57**	Juncin ZI		[37]
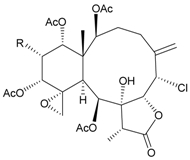	**58**	Juncin ZII (R = OC(O)(CH_2_)_2_CH(CH_3_)_2_)	medium antifeedant activity (84.5%) toward the second-instar larvae of *Spodoptera litura* at a concentration of 500 µg/mL	[38]
			medium cytotoxicity (cell mortality: 20.5% in 24 h and 43.2% in 48 h) toward the second-instar larvae of *S. litura* at a concentration of 100 µg/mL	

*^a^* K562 (human erythromyeloblastoid leukemia), A549 (human lung adenocarcinoma), Hela (human cervical epitheloid carcinoma), Hep2 (human liver carcinoma).

In biological activity testing, juncins R–ZII (**48**–**58**) showed potent antifouling activities against the larval settlement of barnacle *Balanus amphitrite* at a nontoxic concentration (Table 5), and the structure–activity relationships have been discussed [37,38]. The potency of these compounds to inhibit larval settlement was increased when the C-16 exocyclic oxymethylene was substituted by a methylene-bearing chlorine atom and decreased when the exocyclic oxymethylene C-16 was esterified or the acetoxymethylene C-16 was oxygenated to become an esterified group. The chain lengths of the ester moieties at C-1, C-12, C-13 and C-14 and the 11,20-epoxy group could also affect the antifouling activities [37,38].

The known briaranes, gemmacolides A, B, and junceellolide D, were also found to exhibit an antifouling activity as potent as that of juncins R–ZII [38], and these three compounds were not cytotoxic towards the K562, A549, Hela and Hep2 cells. In addition, all the known briaranes showed medium antifeedant activity toward the second-instar larvae of *Spodoptera litura* at a concentration of 500 µg/mL [38].

The gorgonian *J. juncea* collected off the Indian Ocean was proven to be a rich source of interesting natural products. The ethyl acetate extract of *J. juncea* exhibited anti-inflammatory activity at concentrations of 30–100 mg/kg body weight, while the oral median lethal dose (LD_50_) for the extract in albino mice was above 1000 mg/kg. The ethyl acetate extract of *J. juncea* also showed antibacterial activities toward *Bacillus subtilis*, *B. pumilis* and *Escherichia coli* [39]. Six new briaranes, juncins I–M (**59**–**63**) [40] and juncenolide B (**64**) [41], a new sphingolipid, (2*R*,3*R*,4*E*)-1,3-dihydroxy-2-[(nonadecanoyl) amino]-octadec-4-ene (**65**) [42] (Table 6), along with four known briaranes, gemmacolides A–C and juncin H [40], were obtained from the gorgonian coral *J. juncea*, collected from Tuticorin Coast of the Indian Ocean.

**Table 6:** The new natural products from *Junceella juncea*-VI.

Structure	No.	Name	Biological Activity	Ref.
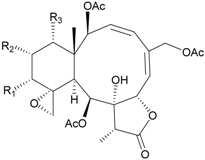	**59**	Juncin I (R_1_ = R_3_ = OAc, R_2_ = OCOCH_2_CH(CH_3_)_2_)	n.r. *^a^*	[40]
	**60**	Juncin J (R_1_ = R_2_ = OCOCH_2_CH(CH_3_)_2_, R_3_ = OAc)	n.r.	[40]
	**61**	Juncin K (R_1_ = R_3_ = OCOCH_2_CH(CH_3_)_2_, R_2_ = H)	n.r.	[40]
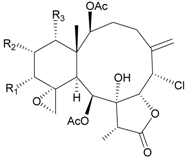	**62**	Juncin L (R_1_ = R_2_ = OCOCH_2_CH(CH_3_)_2_, R_3_ = OAc)	n.r.	[40]
	**63**	Juncin M (R_1_ = R_3_ = OCOCH_2_CH(CH_3_)_2_, R_2_ = H)	n.r.	[40]
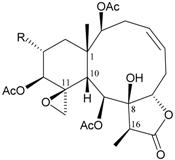	**64**	Juncenolide B (R = OCOCH_2_CH(CH_3_)_2_)	n.r.	[41]
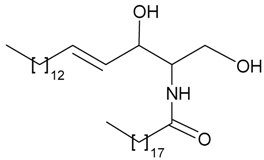	**65**	(2*R*,3*R*,4*E*)-1,3-dihydroxy-2-[(nonadecanoyl) amino]-octadec-4-ene	n.r.	[42]

*^a^* n.r. = not reported.

The molecular formula of juncenolide B was reported as C_30_H_42_O_11_ (M.W. = 578), but the structure presented in the article was found to possess the molecular formula C_30_H_42_O_12_ (M.W. = 594). The spectral data (such as from NOESY experiments) was not sufficient to support the structure presented in the article. We therefore suggested that the structure of this compound (juncenolide B) should be reexamined [41].

Sixteen new briaranes, juncenolides E–K (**66**–**72**) [43,44,45], juncin N (**73**) [46], and junceols A–H (**74**–**81**) [20,47] (Table 7), and two known briaranes, junceellolides B and C, were isolated from the gorgonian *J. juncea*, collected off the waters of Taiwan. Juncenolide G (**68**) is the first naturally-occurring briarane found to have an ether linkage between C-5/C-8 [44], and juncin N (**73**) is the first briarane derivative found to contain a carboxylic group [46]. 

**Table 7:** The new natural products from *Junceella juncea*-VII.

Structure	No.	Name	Biological Activity	Ref.
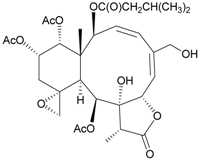	**66**	Juncenolide E	n.r. ^a^	[43]
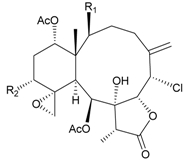	**67**	Juncenolide F (R_1_ = OC(O)CH(CH_3_)_2_, R_2_ = OC(O)CH_2_CH(CH_3_)_2_)	n.r.	[44]
	**69**	Juncenolide H (R_1_ = R_2_ = OAc)	modestly anti-inflammatory	[45]
	**70**	Juncenolide I (R_1_ = OC(O)CH(CH_3_)_2_, R_2_ = OAc)	weakly anti-inflammatory	[45]
	**71**	Juncenolide J (R_1_ = OAc, R_2_ = OC(O)CH_2_CH(CH_3_)_2_)	not active in anti-inflammatory bioassay	[45]
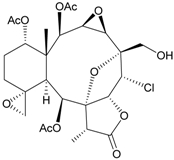	**68**	Juncenolide G	n.r.	[44]
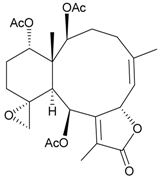	**72**	Juncenolide K	weakly anti-inflammatory	[45]
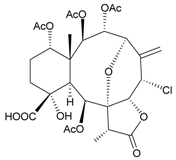	**73**	Juncin N	not active in cytotoxicity testing with P-388D1, DLD-1, IMR-32, RPMI 7951 and CCRF-CEM cells ^b^	[46]
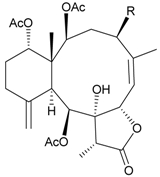	**74**	Junceol A (R = OC(O)CH_2_CH(CH_3_)_2_)	significantly	[20]
			anti-inflammatory	
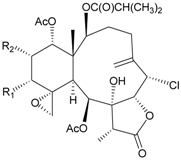	**75**	Junceol B (R_1_ = OAc, R_2_ = OC(O)CH_2_CH(CH_3_)_2_)	significantly	[20]
			anti-inflammatory	
	**76**	Junceol C (R_1_ = R_2_ = OC(O)CH_2_CH(CH_3_)_2_)	significantly	[20]
			anti-inflammatory	
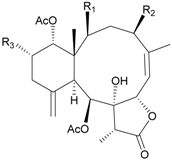	**77**	Junceol D (R_1_ = OC(O)CH(CH_3_)_2_, R_2_ = OC(O)CH_2_CH(CH_3_)_2_, R_3_ = OAc)	not active in anti-inflammaory bioassay	[47]
			exhibited cytotoxicity toward CCRF-CEM and DLD-1 (IC_50_ = 1.3, 10.0 µg/mL) cells	
	**78**	Junceol E (R_1_ = OC(O)CH(CH_3_)_2_, R_2_ = OAc, R_3_ = H)	weakly anti-inflammatory	[47]
			not active in cytotoxicity testing with CCRF-CEM and DLD-1 (IC_50_ > 40 µg/mL) cells	
	**79**	Junceol F (R_1_ = OC(O)CH(CH_3_)CH_2_CH_3_, R_2_ = OAc, R_3_ = H)	moderately anti-inflammatory	[47]
			exhibited cytotoxicity toward CCRF-CEM (IC_50_ = 4.9 µg/mL) cells	
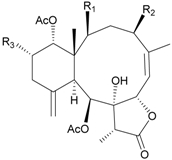	**80**	Junceol G (R_1_ = OC(O)CH(CH_3_)CH_2_CH_3_, R_2_ = H, R_3_ = OAc)	weakly anti-inflammatory	[47]
			exhibited cytotoxicity toward CCRF-CEM (IC_50_ = 4.4 µg/mL)	
	**81**	Junceol H (R_1_ = OAc, R_2_ = H, R_3_ =OC(O)CH(CH_3_)_2_)	weakly anti-inflammatory	[47]
			exhibited cytotoxicity toward CCRF-CEM and DLD-1 (IC_50_ = 7.2, 17.0 µg/mL) cells	

*^a^* n.r. = not reported. *^b^* P388D1 (mouse lymphoid neoplasm), DLD-1 (human colon adenocarcinoma), IMR-32 (human neuroblastoma), RPMI 7951 (human malignant melanoma), CCRF-CEM (human T-cell acute lymphoblastic leukemia).

## 3. Conclusions

**Figure 1:**
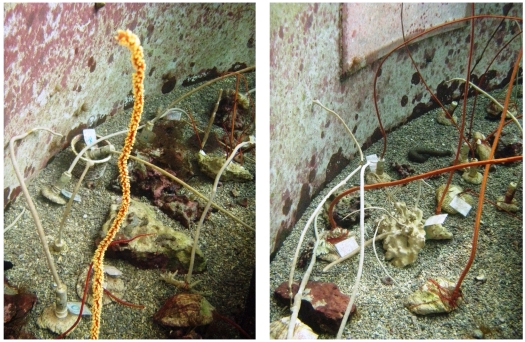
The cultured-type gorgonian corals *Junceella fragilis* (white) and *Junceella juncea* (red).

The chemical class distribution of the natural products obtained from the organisms *Junceella fragilis* and *Junceella juncea* compiled in this review indicates that terpenoid derivatives, particularly briarane-type diterpenoids, are the major components of the natural products isolated. Of the 81 new metabolites, 74 compounds are briarane-type diterpenoids (91.4%). Of these briaranes, over 50% are chlorinated briaranes (38/74 = 51.4%), which are rarely found. Briarane-type compounds continue to attract attention owing to their structural novelty, complexity and interesting bioactivities, such as anti-inflammatory activity [48,49,50,51]. Terpenoid compounds are often present in large amounts in marine invertebrates, and as a major class represent the largest percentage of natural products isolated from marine organisms [52]. Over 500 naturally-occurring briarane derivatives have been isolated from various marine organisms [48,49,50,51]. However, owing to their structural complexity, it is difficult to obtain sufficient amounts of the bioactive metabolites, such as junceols B (**75**) and C (**76**), for further study of their potential medicinal usage. We have therefore begun to culture the potential useful gorgonian corals *J. fragilis* and *J. juncea* (Figure 1) in tanks using our highly developed aquaculture technology for extraction of natural products to establish a stable supply of bioactive materials, which also protects the natural population and habitats from over-exploitation.
